# Antibioprophylaxis in Prevention of Endophthalmitis in Intravitreal Injection: A Systematic Review and Meta-Analysis

**DOI:** 10.1371/journal.pone.0156431

**Published:** 2016-06-03

**Authors:** Cédric Benoist d’Azy, Bruno Pereira, Geraldine Naughton, Frédéric Chiambaretta, Frédéric Dutheil

**Affiliations:** 1 University Hospital of Clermont-Ferrand (CHU), Ophthalmology, Clermont-Ferrand, France; 2 University Hospital of Clermont-Ferrand (CHU), Preventive and Occupational Medicine, Clermont-Ferrand, France; 3 University Hospital of Clermont-Ferrand (CHU), Clinical Research Direction, Clermont-Ferrand, France; 4 Australian Catholic University, Faculty of Health, Melbourne, Victoria, Australia; 5 Clermont Auvergne University, Blaise Pascal University, Laboratory of Metabolic Adaptations to Exercise in Physiological and Pathological conditions EA3533, Clermont-Ferrand, France; 6 Research Centre in Human Nutrition (CRNH) Auvergne, Clermont-Ferrand, France; 7 CNRS, UMR 6024, Physiological and Psychosocial Stress, LAPSCO, University Clermont Auvergne, Clermont-Ferrand, France; International University of Health and Welfare, JAPAN

## Abstract

Despite endophthalmitis being the most feared complication, antibioprophylaxis remains controversial in intravitreal injections. Therefore, we conducted a systematic review and meta-analysis on the effects of antibioprophylaxis in intravitreal injections in the prevention of endophthalmitis. The PubMed, Cochrane Library, Embase and Science Direct databases were searched for studies comparing groups with and without antibiotics in intravitreal injection, with the use of the following keywords: "antibiotic*", "endophthalmitis" and “intravitreal injection*”. To be included, studies needed to specify number of participants and number of endophthalmitis within each group (with and without antibiotics). We conducted meta-analysis on the prevalence of clinical endophthalmitis including both culture-proven and culture negative samples. Nine studies were included. A total of 88 incidences of endophthalmitis were reported from 174,159 injections (0.051% i.e., one incidence of endophthalmitis for 1979 injections). Specifically, 59 incidences of endophthalmitis were reported from 113,530 injections in the group with antibiotics (0.052% or one incidence of endophthalmitis for 1924 injections) and 29 incidences of endophthalmitis from 60,633 injections in the group without antibiotics (0.048% or one endophthalmitis for 2091 injections). Our meta-analysis did not report a significant difference in the prevalence of clinical endophthalimitis between the two groups with and without topical antibiotics: the odds ratio of clinical endophthalimitis was 0.804 (CI95% 0.384–1.682, p = 0.56) for the antibiotic group compared with the group without antibiotics. In conclusion, we performed the first large meta-analysis demonstrating that antibioprophylaxis is not required in intravitreal injections. Strict rules of asepsis remain the only evidence-based prophylaxis of endophthalmitis. The results support initiatives to reduce the global threat of resistance to antibiotics.

## Introduction

Anti-VEGF intravitreal injections have demonstrated a relative efficacy to treat common diseases such as wet age-related macular degeneration, diabetic macular edema and central and branch retinal vein occlusion [1]. Therefore, intravitreal injections are a common medical procedure, performed annually by millions across industrialized countries [2]. Transfixing perforation of the sclera of the eye by the injection needle might lead to infectious complications, specifically endophthalmitis [3]. Although rare [4,5,6,7], endophthalmitis is the most feared complication of intravitreal injections due to its poor functional prognosis [8]. Strategies for infection prophylaxis are generally based on the use of antiseptics and antibiotics. Regarding antiseptics, the use of povidone-iodine to the ocular surface along with aseptic techniques in anterior segment surgery have been the only type of intervention shown to be moderately important in reducing the risk of post-operative endophthalmitis [9]. However, the use of antibiotics remains under debate. Despite the absence of evidence-based data, the common clinical practice involved topical antibiotics before, concurrently with, or after the intravitreal injection [10]. The usefulness of antibiotics in the prevention of endophthalmitis after intravitreal injections has been more recently questioned. No prospective randomized study demonstrated the benefits of topical antibiotics. Additionally, topical antibiotics enhanced rapid development of antibiotic-resistant virulent bacteria on the ocular surface and spreading in the oral sphere [11,12].

Despite the absence of data to support the reduction of endophthalmitis through the use of antibiotics after intravitreal injection, many ophthalmologists continue to recommend a multiday course of topical antibiotic use after intravitreal injection. Therefore, because antibiotics can be unecessary and detrimental [13], we aimed to conduct a systematic review and meta-analysis on putative benefits of antibiotic prophylaxis in preventing endophthalmitis after intravitreal injections, and to strengthen the evidence base for establishing international guidelines.

## Methods

### Literature Search

**Fig 1:**
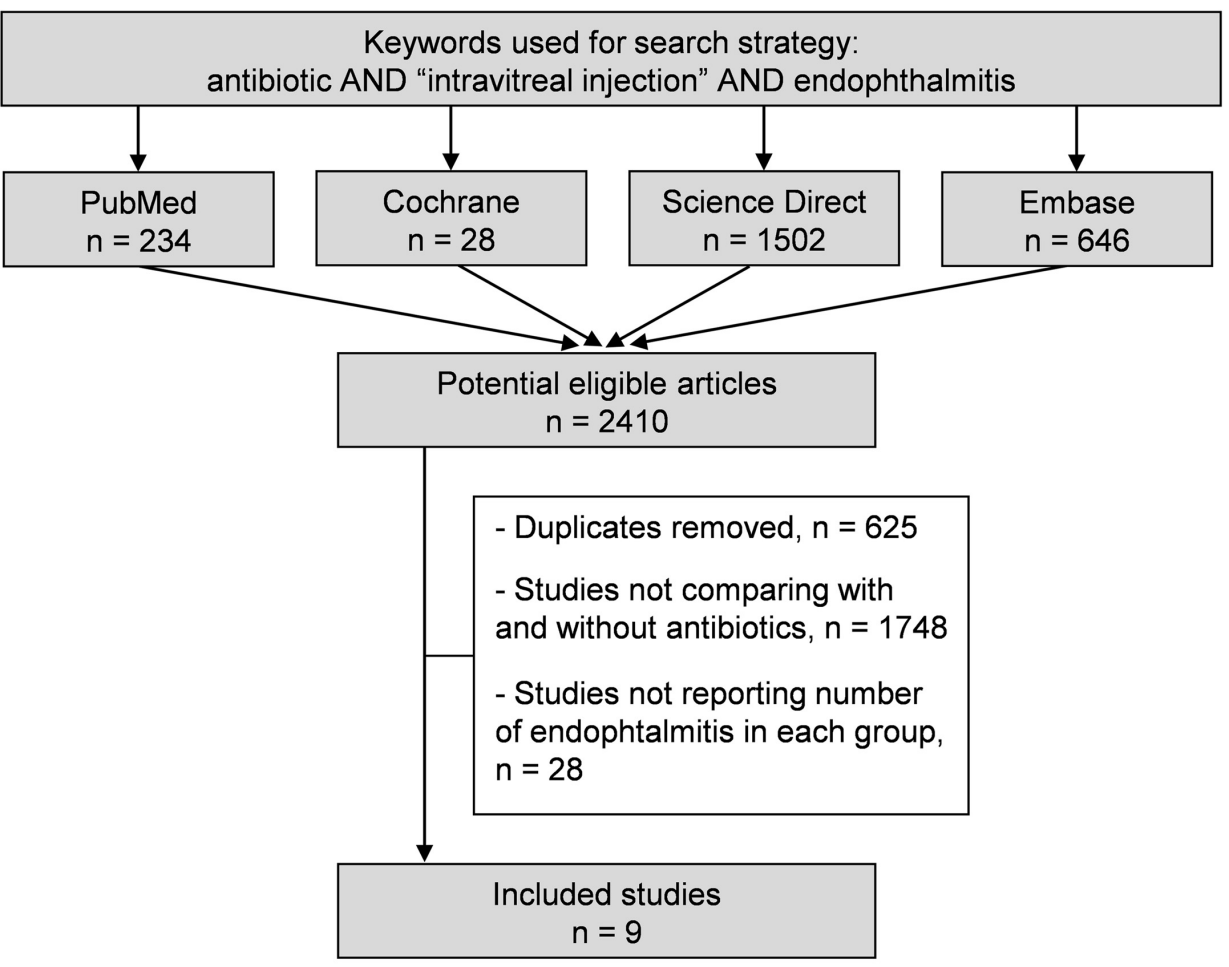
Search strategy.

We reviewed all cohort studies found on Medline via Pubmed that compared two groups after intravitreal injections: one with antibiotics and one without. Keywords for the search strategy were "antibiotic*", "endophthalmitis" and “intravitreal injection*”. The following databases were searched on December 23^rd^, 2015: PubMed, Cochrane Library, Science Direct and Embase. The search was not limited to specific years and no language restrictions applied. To be included, studies needed to specify the number of participants and quantify the incidence of endophthalmitis within each group (with and without antibiotics). All kinds of intravitreal injections were included i.e. corticoids (triamcinolone, dexamethasone) or anti-VEGF such as monoclonal antibodies (mab–bevacizumab, ranibizumab), recombinant proteins (aflibercept) and pegatnamib. In addition, reference lists of all publications meeting the inclusion criteria were manually searched to identify any further studies not identified via electronic searching. The search strategy is described in Fig 1. One author (CBA) conducted all literature searches and collated the abstracts. Two authors (CBA and FD) separately reviewed the abstracts and based on the selection criteria, decided the suitability of the articles for inclusion. A third author (GN) was asked to review the article where consensus on suitability was not met. Then, all authors reviewed the eligible articles.

### Quality of assessment

Although not designed for quantifying the integrity of studies [14], the “STrengthening the Reporting of OBservational studies in Epidemiology” (STROBE) criteria were used for checking the quality of reporting [15]. The 22 items identified in the STROBE criteria could gain achieve a maximal score of 33.

### Statistical considerations

Statistical analysis was conducted using Comprehensive Meta-analysis software (version 2, Biostat Corporation) and Stata software (version 13, StataCorp, College Station, US). Extracted data were summarized for each study and reported as mean (standard-deviation) and number (%) for continuous and categorical variables respectively. Prevalence of endophthalmitis and 95% confidence intervals were estimated using random-effects models assuming between and within study variability (DerSimonian and Laird approach). Statistical heterogeneity between results was assessed using forest plots, confidence intervals and I². The I^2^ statistic is the most common metric for measuring the magnitude of between-study heterogeneity and is easily interpretable. I² values range between 0 and 100% and are typically considered low for values <25%, modest for 25–50%, and high for >50%. This statistical method generally assumes heterogeneity when the p-value of the I² test is <0.05. A sensitivity analysis was thus conducted to assess the influence on the global prevalence of the inclusion and exclusion of studies. Then, we conducted a meta-analysis on the prevalence of clinical endophthalmitis including both culture-proven and culture negative samples and where possible (sufficient sample size), on the prevalence of endophthalmitis depending on the type of products used for intravitreal injections (taking into account antibiotics prescription), and on the type of germs identified if the culture was positive. We also aimed to propose meta-regressions too investigate relations between the prevalence and clinically relevant parameters according to the literature. Results were expressed as regression coefficients and 95% confidence intervals. Type I-error was fixed at a = 0.05.

## Results

An initial search produced a possible for 943 articles. Removal of duplicates and selection criteria reduced the articles comparing two groups to nine articles (Fig 1 and Table 1).

**Table 1:** Repartition of culture in the 2 groups in each study and prevalence of endophthalmitis in each study.

Study	Total			Group with antibiotic			Group without antibiotic		
	Number of endophthalmitis / Number of injections	Culture (+)	Culture (-)	Number of endophthalmitis / Number of injections	Culture (+)	Culture (-)	Number of endophthalmitis / Number of injections	Culture (+)	Culture (-)
Bhatt et al, 2011	10/4767	1	9	5/2287	0	5	5/2480	1	4
Bhavsar et al, 2012	7/8027	5	2	6/4697	4	2	1/3333	1	0
Cheung et al, 2012	9/15895	3	6	7/10629	1	6	2/5266	2	0
Meredith et al, 2015*	11/18509	7	3	8/16509	6	1	3/2000	1	2
Pachuo et al, 2015	0/620	0	0	0/310	0	0	0/310	0	0
Park et al, 2013	3/17332	1	2	0/8649	0	0	3/8683	1	2
Ramel et al, 2014	6/11450	5	1	3/10144	2	1	3/1306	3	0
Storey et al, 2014**	39/92554	14	25	28/57654	10	18	11/34900	4	7
Stranak et al, 2014	3/5005	1	2	2/2651	0	2	1/2355	1	0
**Total**									
Number	88/174159	37	50	59/113530	26	35	29/60633	14	15
Prevalence (%)		0.021	0.029		0.020	0.031		0.023	0.018

* 1 endophthalmitis had not culture results

** Transition period with unknown antibiotic prescription was excluded

### Quality of articles

Quality assessment of the nine included studies was analyzed by the STROBE criteria, from which results varied from 57.6 to 94.0%, with a mean score of 77.1±13.7%. Overall, the studies performed best in the discussion section and worst in the methods section.

### Population

#### Sample size

Only two studies reported the number of patients i.e. 4767 [16] and 1185 participants [17]. All studies reported the total number of injections ranging from 620 [18] to 92,554 [19]. Overall, our current meta-analyses included a total of 174,159 injections: 113,530 with antibiotics and 60,633 without antibiotics (Table 1).

#### Age

Only two studies reported the median age within patients; specifically 72.2 [13] and 79.3 [20] years old. A third study reported an age of the population ranging from 30 to 70 years old, but did not specify a mean or median age [18]. Other studies did not provide any information on the age of patients.

#### Gender

Gender was not specified in all of the nine retrieved studies.

### Intravitreal injections

#### Indications for injections

Four studies provided details of the indications for injections which were exudative age-related macular degeneration [13,17,18], choroidal neovascular membranes secondary to myopic degeneration [13,18], macular edema secondary to diabetes [13,18,21], central and branch retinal vein occlusions [13,18], uveitis [13,18], birdshot chorioretinopathy [13] and other proliferative retinopathies [13]. The five other studies included all patients needing intravitreal injections but did not specify more detailed indications [16,19,20,22,23].

#### Type of products injected

all studies reported injections of bevacizumab except one [21], all studies reported injections of ranibizumab except one [18], six studies used triamcinolone injections [13,16,20,21,22,23], three studies reported pegatnamib injections [13,20,23], one gave aflibercept [19], one reported dexamethasone injections [23] and one reported a C3F8 injection [22]. Among the studies reporting multiple products within the injection, five combined injections of bevacizumab, ranibizumab and triamcinolone [13,16,20,22,23], three combined bevacizumab, ranibizumab, triamcinolone and pegatnamib [13,20,23], one reported simultaneously ranibizumab and triamcinolone [21], and one bevacizumab and ranibizumab [19]. However, only one of these studies reported the sample size with and without antibiotics within each group of type of products injected [19]. Subsequently, more advanced meta-analyses were unable to be conducted.

### Antibiotics use

All included articles reported only topical antibiotics, with the exception of one study which also administrated one oral dose of antibiotic post injection (ciprofloxacin 500 mg) during three days in addition to topical antibiotics [18]. One study prescribed topical antibiotics only for the three days *preceding the* intravitreal injection [22]. Another study used a topical antibiotic *before and after the injection* (levofloxacin 5 times daily the 3 days before and the 3 days after injection) [23]. Four studies administered antibiotics *during and after* the injection (one drop immediately at the end of procedure and then a 4^th^ generation fluoroquinolone after 3 or 5 days [13,16] or tobramycin 4 times daily during 4 days [20] or azithromycin twice daily during 3 days [20] or others left the prescription of topical antibiotics to the discretion of individual physicians). Finally, one article reported antibiotics only *after* the injection (4 times daily during 4 days) [19]. Two articles left the prescription of topical antibiotics to the discretion of physicians, and thus did not specify whether antibiotics were administered before, during or after intravitreal injection [17,21]. One study reported a transition period in which the antibiotic prescription was unknown; excluding this transition period from our meta-analyses [19].

### Bacteriology

*Staphylococcus* and *streptococcus* are the most frequent germs found in culture-proven endophthalmitis (47.5% and 35%, respectively) (Table 2). Enterococcus was found within two studies [13,19], and only one lactobacillus was found out of 37 culure-proven endophthalmitis [19].

**Table 2:** Bacteria found in culture proven endophthalmitis.

Study	Culture	Group with antibiotic				Group without antibiotic			
	(+)	Staphylococcus	Streptococcus	Enterococcus	lactobacillus	Staphylococcus	Streptococcus	Enterococcus	lactobacillus
Bhatt et al, 2011	1	0	0	0	0	0	1	0	0
Bhavsar et al, 2012	5	3	1	0	0	1	0	0	0
Cheung et al, 2012	3	0	1	0	0	0	1	1	0
Meredith et al, 2015	7	4	2	0	0	0	1	0	0
Pachuo et al, 2015	0	0	0	0	0	0	0	0	0
Park et al, 2013	1	0	0	0	0	1	0	0	0
Ramel et al, 2014	5	2	0	0	0	3	0	0	0
Storey et al, 2014*	14**	3	4	2	1	1	2	0	0
Stranak et al, 2014	1	0	0	0	0	1	0	0	0
**Total**	37	12	8	2	1	7	5	1	0

* Transition period with unknown antibiotic prescription was excluded

** 1 culture was non differentiated gram-positive cocci

### Outcomes and aims of the studies

The primary outcome was the incidence of endophthalmitis between two groups: one with antibiotics and one without. Secondary outcomes were clearly defined in two studies and involved investigations of the microbial spectrum [13,17,19] and clinical outcomes including return to baseline visual acuity [17,19]. However, other studies also described microbial spectrum [16,18,20,21,22,23] and four studies reported the number of endophthalmitis for each product used [13,19,22,23]. However, whether it was in group with or without antibiotics was unclear.

### Study designs

Six studies were retrospective [13,16,19,20,22,23] and three were prospective [17,18,21]. All studies were published after 2011; with the duration of follow-up ranging from 12 [18] to 108 months [21].

### Meta-analysis on prevalence of clinical endopthalmitis

**Fig 2:**
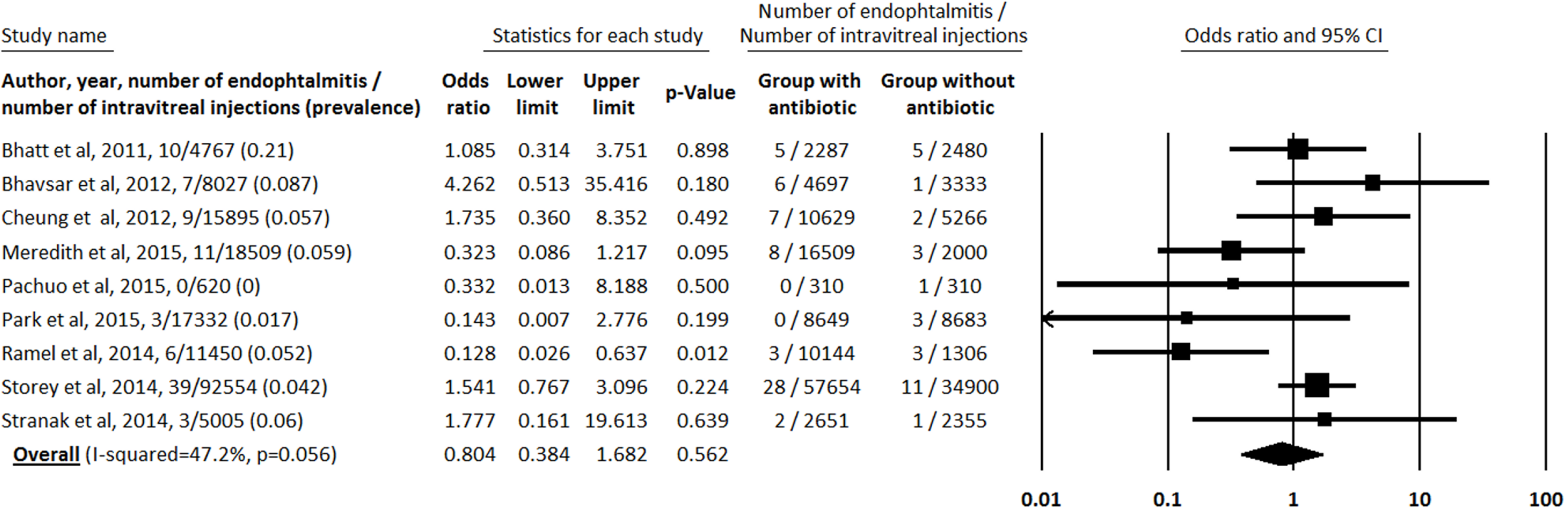
Odd ratio for each study including both culture-proven and culture negative endophthalmitis.

A total of 88 incidences of endophthalmitis were reported from the 174,159 injections (0.051% or one incidence of endophthalmitis from 1979 injections), ranging from 0/620 (0%) [18] to 1/477 (0.21%) [16]. Specifically, 59 endophthalmitis were reported from 113,530 injections in the group with antibiotics (0.052% or one incidence of endophthalmitis from 1924 injections) and 29 endophthalmitis from 60,633 injections in the group without antibiotics (0.048% or one incidence of endophthalmitis from 2091 injections) (Table 1). Our meta-analysis did not report a significant difference in the prevalence of clinical endophthalimitis between the two groups with and without topical antibiotics: the odds ratio of clinical endophthalimitis was 0.804 (CI95% 0.384–1.682, p = 0.56) for the antibiotics group compared with the group without antibiotics, with moderate heterogeneity between studies (I-squared = 47.2%, p = .056) (Fig 2).

### Meta-analysis on prevalence of culture-proven endopthalmitis

**Fig 3:**
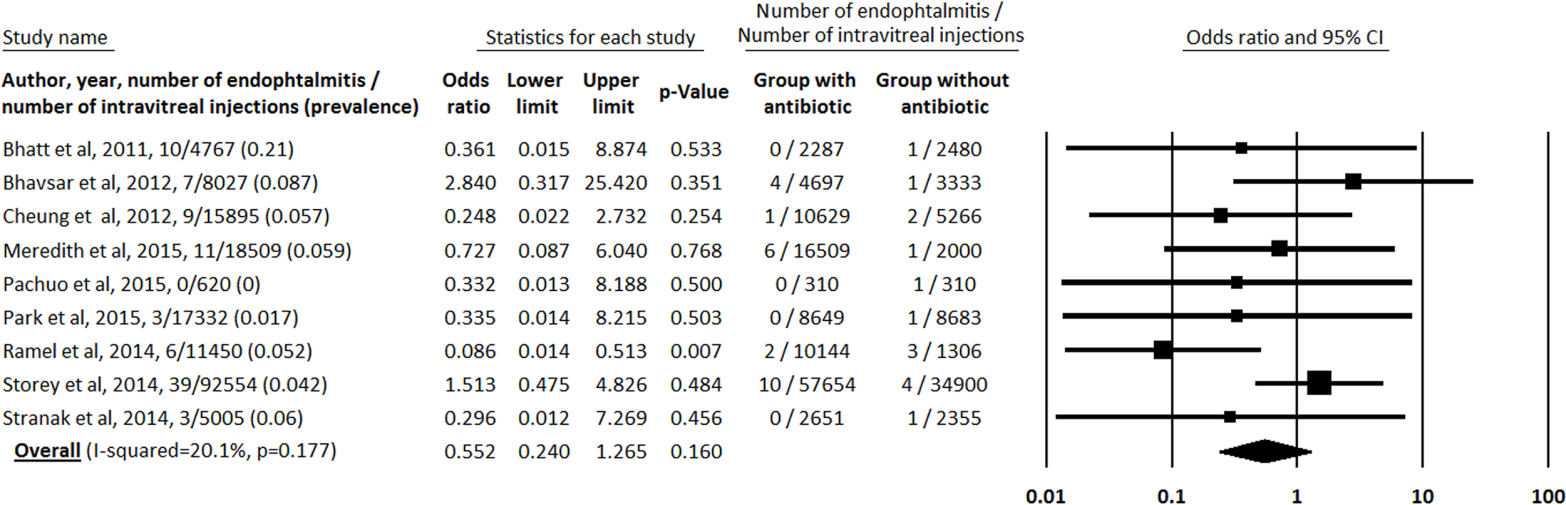
Odd ratio for each study including only culture-proven endophthalmitis.

When considering only culture-proven endophthalmitis, a total of 37 incidences of culture-proven endophthalmitis were reported from 174,159 injections (0.021% or one incidence of endophthalmitis from 4,707 injections) and thus, a total of 50 culture-negative cases of suspected endophthalmitis were reported from 174,159 injections (0.029% or one incidence of endophthalmitis from 3,483 injections) (one described case of endophthalmitis lacked culture results) (Table 1) [17]. Specifically, 23 culture-proven endophthalmitis were reported from 113,530 injections in the group with antibiotics (0.020% or one incidence of endophthalmitis from 4,936 injections) and 14 incidences of culture-proven endophthalmitis from 60,633 injections in the group without antibiotics (0.023% ior one incidence of endophthalmitis from 4,331 injections). Including only culture-negative cases, 35 culture-negative incidences of endophthalmitis were reported from 113,530 injections in the group with antibiotics (0.031% or one incidence of endophthalmitis from 3,244 injections) and 15 incidences of culture-proven endophthalmitis from 60,633 injections in the group without antibiotics (0.025% or one incidence of endophthalmitis from 4,042 injections). Our meta-analysis did not report a significant difference in the incidence of culture-proven endophthalimitis between the 2 groups with and without topical antibiotics: the odd ratio of culture-proven endophthalimitis was 0.552 (CI 95% 0.240–1.265, p = 0.160) for the antibiotics group compared with the group without antibiotics; with low heterogeneity between studies (I-squared = 20.1%, p = 0.177) (Fig 3).

### Other meta-analyses and meta-regressions

An insufficient number of studies reported the specific types of antibiotics used and types of products injected (bevacizumab, ranibizumab, triamcinolone). Therefore, low numbers of studies precluded meta-analyses on the influence of types of products injected to reduce the risk of endophthalmitis. Insufficient sample sizes also precluded meta-analyses on bacteries retrieved in culture proven endophthalmitis. Similarly, only two studies reported the number of patients, only two studies reported age, and no studies reported gender (S1 Database). Consequently, no meta-regressions were conducted.

## Discussion

This current study is the first large meta-analysis on the effectiveness of antibioprophylaxis in the prevention of endophthalmitis via intravitreal injection. We included a total of 174,159 injections, with a 0.052% or one incidence of endophthalmitis from 1924 injections) in the group with antibiotics and 0.048% or one incidence of endophthalmitis from 2091 injections in the group without antibiotics. The major finding ere was the absence of difference in the incidence of endophthalmitis with and without antibioprophylaxis (OR = 0.804, CI95% 0.384–1.682, p = 0.56) for the antibiotic group compared with the group without antibiotics; with a moderate heterogeneity between studies (I-squared = 47.2%, p = .056).

### The threat of antibiotics

Repeated use of antiobioprophylaxis, such as in monthly intravitreal injections, promote resistance [11] and virulence [24] of the conjunctival flora, even on short durations with low doses [24]. The minimum inhibitory concentration of antibiotics at the conjunctival flora increased in patients receiving antibiotics after the third injection [11]. Moreover, topical antibiotics and povidone–iodine before intravitreal injections did not further reduce bacterial concentrations compared with povidone–iodine alone [25]. Therefore, some authors calculated the sample size required to perform a non-inferiority test of antiseptics compared with antibiotics, ranged from 25,000 [13] to 50,000 [20] injections per group. In addition, a single application of povidone–iodine demonstrated a bactericidal effect equivalent to a 3-day course of topical antibiotics [26]. Also, repeated topical antibiotics applications may cause resistance in the nasopharynx [27]. Pharmacologically, intravitreal penetration of topical antibiotic is below the minimum inhibitory concentration required for a therapeutic effect [28]. Finally, systematic use of topical antibiotics generates a substantial cost, both for patients and society. The evidence-based strategy for preventing endophthalmitis remains the use of an eyelid speculum, povidone-iodine, surgical mask and sterile gloves [29].

### Promoting guideline for antibioprophy

This is the first large meta-analysis demonstrating the absence of systematic antibioprophylaxis in prevention of endophthalmitis in intravitreal injections. The moderate heterogeneity reported in our study is due to the low incidence of endophthalmitis; ranging from 0 [18] to 0.21% [16]. Even if the study of Dorssaps et al (2015) could not have been included in our meta-analyses because it did not report numbers of endophthalmitis and total number of intravitreal injections within a group with and without antibiotics, authors reported that the use of an antibiotic or antiseptic increased the incidence of endophthalmitis (relative risk = 2.77, CI95% 1.54–5.00, p = .001) [4]. Moreover, we must acknowledge that the study of Dorssaps et al (2015) was conducted over 25 centers and included more than 300,000 intravitreal injections [4]. Therefore, despite the absence of guidelines regarding use the antibiotics in intravitreal injections, some ophthalmologists recently stopped antibioprophylaxis. Also some ophthalmologists reported a low incidence of endophthalmitis can be achieved in the absence of topical antibiotics [30]. Moreover, one of the lowest incidences of endophthalmitis in the literature was reported in this study that included a large sample size of intravitreal injections without antibiotics; resulting in only one incidence of endophthalmitis from 18,839 injections (0.0053%) [30].

### Limitations

All meta-analyses have limitations [31]. Meta-analyses inherit the limitations of the individual studies of which they are composed. The low number of prospective studies reported the lowest number of injections. Antibiotic treatment protocols differed between selected studies and insufficient data precluded further analyses on protocols used. The incidence of culture-negative endophthalmitis may seem high however, it is similar to literature (0.022%) [5]. Even if non-significant, the culture-proven endophthalimitis odds ratio of 0.552 (CI 95% 0.240–1.265, p = 0.160) for the antibiotics group compared with the group without antibiotics may limit generalizability. However, in real practice, all endophthalmitis generate an undesirable prognosis (including culture negative endophthalmitis) [19]. A large multicenter prospective cluster randomized controlled trial, with consistent protocols, would help promote international guidelines; nonetheless the sample size required would make this study problematic to manage. Preliminary plans to target sub-group analyses for types of antibiotic used, products injected, and the bacteria retrieved in culture-proven endophthalmitis, were not feasible due to insufficient numbers of studies with these details.

## Conclusion

We performed the first large meta-analysis demonstrating that antibioprophylaxis treatment is not required in intravitreal injections. Strict rules of asepsis remain the only evidence-based support for prophylaxis of endophthalmitis. The results support initiatives to reduce the global threat of resistance to antibiotics. Therefore, antibiotics should be prescribed only in exceptional cases such as immunosuppression or fragile conjunctiva. International guidelines surrounding the use of antibiotics in intravitreal injections should be generated.

## Supporting Information

S1 PRISMA Checklist PRISMA Checklist. (DOCX)

S1 Database Crude data of all the included studies. Titles of columns are written without abbreviations.
(XLSX)
